# Influence of different surface treatment on bonding of metal and ceramic Orthodontic Brackets to CAD-CAM all ceramic materials

**DOI:** 10.1186/s12903-023-03246-x

**Published:** 2023-08-13

**Authors:** Satheesh B. Haralur, Abdulrahman Mushabbab Alqahtani, Abdullah Saeed Shiban, Zyad Muhammed Alattaf, Saurabh Chaturvedi, Saeed M AlQahtani, Nasser M Alqahtani

**Affiliations:** 1 Department of Prosthodontics, College of Dentistry, King Khalid University, Abha, 62529 Saudi Arabia; 2 Interns, College of Dentistry, King Khalid University, Abha, 62529 Saudi Arabia

**Keywords:** Orthodontic brackets, CAD-CAM all ceramic, Surface treatment protocols, Lithium disilicate, ), polymer infiltrated ceramic, Zirconia-reinforced lithium silicate glass ceramic, 5YTZP zirconia, Shear bond strength

## Abstract

**Background:**

Developing efficient bonding techniques for orthodontic brackets and all-ceramic materials continues to pose a clinical difficulty. This study aimed to evaluate the shear bond strengths (SBS) of metal and ceramic brackets to various all-ceramic CAD-CAM materials, such as lithium disilicate CAD (LDS-CAD), polymer-infiltrated ceramic (PIC), zirconia-reinforced lithium silicate glass ceramic (ZLS), and 5YTZP zirconia after different surface treatments and thermal cycling.

**Materials and methods:**

The samples were divided into two groups to be bonded with ceramic and metal lower incisor brackets. Each group was subdivided into a control group devoid of any surface treatment, 10% HF acid (HFA) etching, ceramic etch & prime (MEP), Al2O3 air abrasion, and medium grit diamond bur roughening. After surface treatment, brackets were bonded with composite resin cement, thermal cycled, and tested for shear bond strength. The failed surfaces were evaluated with a digital microscope to analyse the type of failure. The data were statistically analysed using a one-way ANOVA and Tukey HSD tests at p < 0.05.

**Results:**

The highest mean bond strengths were found with HFA etching in LDS-CAD (13.17 ± 0.26 MPa) and ZLS (12.85 0.52 MPa). Diamond bur recorded the lowest mean bond strength roughening across all the ceramic groups. There were significant differences in mean shear bond values per surface treatment (p < 0.001) and ceramic materials.

**Conclusion:**

Among the surface treatment protocols evaluated, HFA etching and MEP surface treatment resulted in enhanced bond strength of both ceramic and metal brackets to CAD-CAM all ceramic materials.

## Background

Progressive advancements in orthodontic technique have led to a substantial number of adult patients seeking orthodontic treatment [1]. Majority of these patients have had their teeth restored with various materials, and restorative treatments are often performed concurrently with orthodontic treatment [2, 3]. As part of orthodontic rehabilitation, the adult patient may require partial or full veneer crowns, such as laminate veneers or jacket crowns. Bonding orthodontic brackets to teeth is a routine approach to tooth alignment in fixed orthodontic appliances [4]. Developing the optimum bond strength between the orthodontic bracket and different restoration surfaces is a clinical challenge during adult orthodontic therapy. Researchers estimated the average force range required for translatory movement and extrusion of teeth to be 70–120 g and 35–60 g, respectively [5]. Hence, Reynolds et al. [6] recommended a minimal bond strength of 6-8 MPa between orthodontic brackets and teeth for clinical orthodontic movement. Hence, the bond strength at the bracket-adhesive-restoration surface interface must withstand orthodontic forces without detachment. Orthodontic brackets are available in metal and ceramic materials. Metal brackets are manufactured from medical-grade stainless steel; many patients dislike them because of the marked colour difference against tooth shades. Due to their similar colour to the tooth, people prefer monocrystalline or polycrystalline alumina ceramic brackets.

Contemporary dentistry considers dental ceramics the most suitable restorative materials due to their biocompatibility, mechanical and aesthetic properties. Advances in computer-aided design (CAD) / computer-aided manufacturing (CAM) technologies have promoted their wide application in modern restorative dentistry. Various CAD-CAM materials are constantly evolving to enhance bio-mechanical and aesthetic parameters to improve long-term clinical performance and patient aesthetic demands. Full contour restoration fabrication from high-strength CAD-CAM materials eliminate the disadvantages of two dissimilar materials, like veneer layer fracture and reduced translucency.

Lithium disilicate (LD-CAD) ceramics have a wide application due to their superior translucency and aesthetic appearance, along with their strong bond strength to tooth substrates [7]. Dissolution of acid-sensitive silica particles facilitated mechanical and chemical adhesion to the tooth substrate [8, 9]. A new generation of 5YTZP zirconia (5 YZP) with a 5 mol% yttria stabilized zirconia polycrystalline ceramic with a 10–50% cubic phase has been developed for anterior aesthetic restorations. It provides a smooth progression of shade and translucency by removing the discernible layer of colour [10].

A new generation of zirconia-reinforced lithium silicate glass ceramic material (ZLS) was developed to achieve the positive material characteristics of zirconia (ZrO2) and glass ceramic. The 10 wt% by weight of ZrO2 and 0.1 wt% lanthanum oxide are added during the nucleation process. This results in enhanced mechanical properties and retains glass ceramics’ brilliant optical properties [11]. In recent years, new-generation ceramics have been developed to utilize ceramic and resin composite features. Resin-ceramics comprised 60–86% inorganic ceramic particles by weight and 40 − 14% polymers [12]. Hybrid polymer infiltrated ceramics (PIC) possess optimum mechanical properties and have a compatible modulus of elasticity with dentin substrates [13].

Various authors evaluated different surface treatment protocols to enhance bracket and dental restoration bond strength. Sarac et al. [14] assessed the effect of air alumina particle air abrasion and tribochemical silica coating on the bond strength of felspathic, fluorapatite, and leucite-reinforced dental ceramic surfaces to brackets. They concluded that the SBS for fluoroapatite ceramic was significantly lower than that for leucite-reinforced ceramic with air particle abrasion, while they recorded a higher SBS value for the silica coating of leucite ceramic surfaces. Zhang ZC et al. [15] evaluated the effects of hydrofluoric acid, silane, alumina sandblasting, and silica-coating applications on the shear bond strengths of metal brackets bonded to a silica-based ceramic. Isolated hydrofluoric acid surface treatment displayed suboptimal SBS for clinical applications. Combining HF acid etching with a silane application has resulted in achieving the highest Shear Bond Strength (SBS) values on IPS Classic ceramics.

The structural composition of CAD/CAM dental ceramics varies. Consequently, the resultant surface morphology is different after physical surface treatments, such as air abrasion, hydrofluoric acid etching, and roughening with a bur. Since PIC, ZLS, and 5 YZP are relatively new in the dentistry market, studies related to their performance in various areas, such as bonding features, are ongoing [16]. This study aimed to examine the effect of various surface treatments on the bond strength of metal and ceramic brackets to different CAD/CAM dental ceramics. A secondary objective was to determine the types of bonding failure by comparing these surface treatments with metal/ ceramic brackets. The null hypotheses were that the various surface treatments would not influence the bond strength and type of failure of metal/ ceramic brackets or CAD/CAM dental ceramic materials.

## Materials and methods

Details of the four ceramic groups evaluated in the study are metioned in the Table 1.Flat surface discs from all the tested groups in the dimensions of 6 mm X 3 mm X 2 mm were prepared by the precision sectioning diamond saw (Isomet 1000; Buehler, Waukegan Road Lake Bluff, IL, USA). LD-CAD specimens were subjected to crystallizing and glazing heat treatments as recommended by the manufacturer. For 5YTZP sintered zirconia crowns, they were polished with the sequential use of 320, 600, and 1200 grit SiC abrasive papers under slow-running water on a rotational polishing device (Jean Wirtz, Charlottenstr 73, Düsseldorf, Germany). The ZLS ceramic discs underwent crystallization and combination firing heat treatment, according to manufacturer guidelines. The discs were then polished using a pre-polished pink diamond-coated cup, followed by high gloss polishing with grey diamond-coated cup instruments.

**Table 1:** CAD-CAM ceramic materials used in the study

Material	Brand	Composition	Manufacturer	Lot no
Lithium Disilicate (Glass Ceramics)	IPS e.max®CAD	SiO_2_^,^ Li_2_O, K_2_O, P_2_O_3_, ZrO_5_, ZnO, Al_2_O_3_, MgO.	*Vita Zahnfabrik, Bad Säckingen, Germany*	X15367
Hybrid polymer infiltrated ceramics	Enamic	*UDMA, TEGDMA, SiO2, Al2O3, Na2O, K2O, B2O3, Zr2O, CaO*	*Vita Zahnfabrik, Bad Säckingen, Germany*	59,882
Zirconia reinforced glass ceramic	Suprinity	SiO_2_, Li_2_O, K_2_O, P_2_O, Al_2_O_3_^,^ ZrO_2_^,^ CeO_2 ,_La_2_O.	*Vita Zahnfabrik, Bad Säckingen, Germany*	58,081
5YTZP zirconia	Cercon xt	DeguDent GmbH, Hanau-wolfgang, Germany.	Dentsply Sirona,	18,043,031

**Fig. 1:**
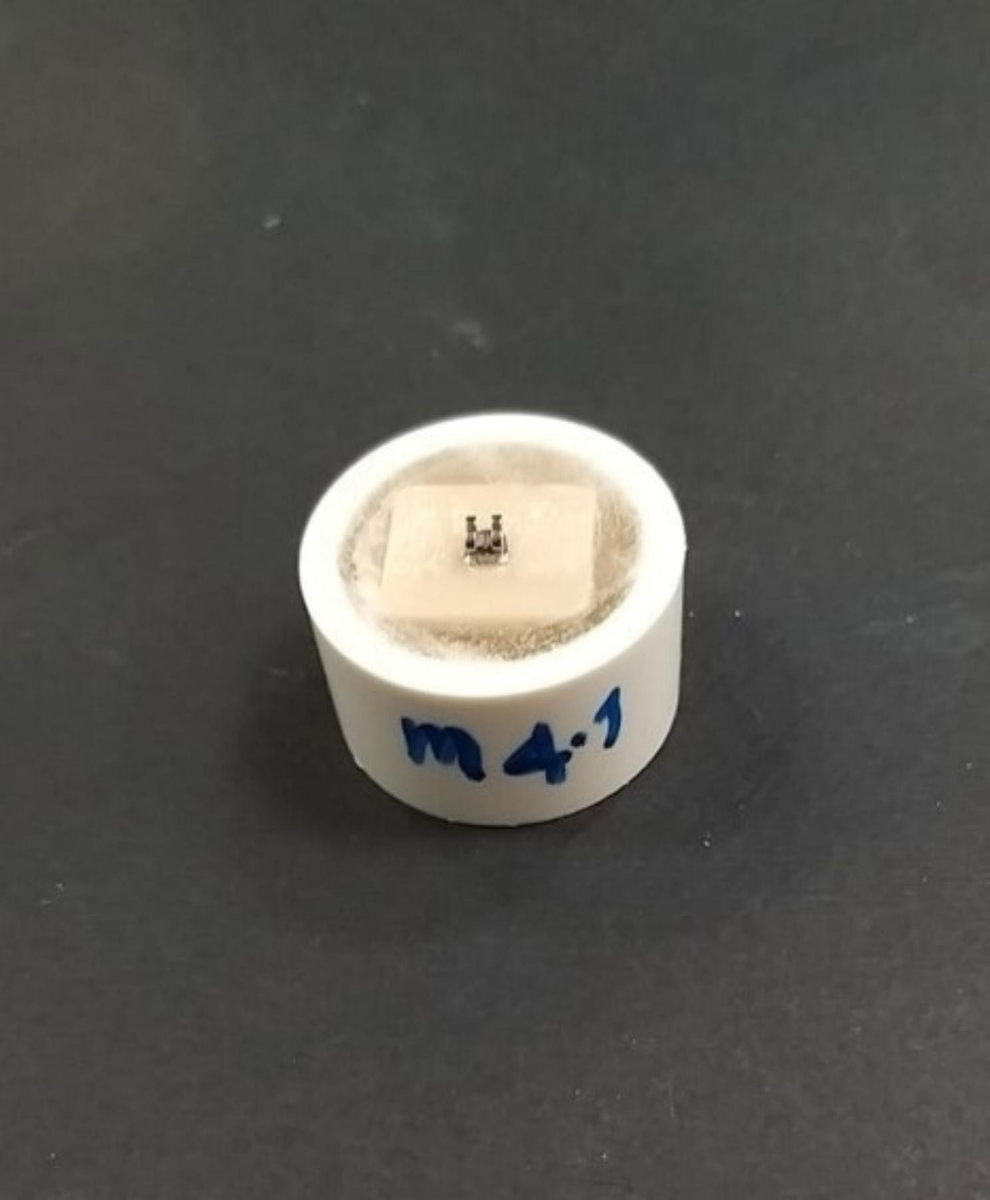
Embedded ceramic samples with bonded metal orthodontic bracket

PIC ceramic discs were processed using SiC instruments rotating at a speed of 7000 RPM/min. Subsequently, high-gloss polishing was performed using a diamond-coated instrument at 5000 RPM/min. The surface polishing of all ceramic discs was standardized by a tactile profilometer (Surftest SJ 201, Mitutoyo, Tokyo, Japan) with an evaluation length of 1.5 mm at 0.5 mm/s. Surface roughness was measured three times for each sample, and the minimum mean roughness value (Ra) was maintained at 0.2 𝜇m for all the ceramic discs [17]. Post-polishing, each ceramic disc was embedded (Figure 1) into a chlorinated poly (vinyl chloride) cylinder with auto-polymerizing clear polymethyl methacrylate acrylic resin (Major. Base.20, Major Prodotti Dentari S.p.A., Moncalieri, Italy).

### Ceramic sample groups

100 samples of each ceramic type were fabricated. They were randomly divided into two main groups (n = 50) to be bonded with metal and ceramic lower incisor orthodontic brackets. Subsequently, each main group was subdivided into 5 subgroups (n = 10) according to the surface treatment. A sample of 10 in each subgroup was estimated following earlier published studies [18, 19]. The sample size calculation was performed using G* Power software (version 3.1; University of Düsseldorf), with an effect size (d) of 1.4, α of 0.05, and 1-β (power) of 0.80[20].

### Surface treatment protocol

#### Control

Polished and finished ceramic samples were bonded with orthodontic brackets with no additional surface treatment.

#### HF acid etching

The duration of etching the finished specimens with 10% HF acid (FGM, Fort Lauderdale FL 33,309, US) varied depending on the type of ceramic. LDS ceramic and ZLS ceramic samples were etched for 20 s, PIC ceramic groups for 60 s, and 5YTZP specimens for 60 min. After the specified etching time, HF acid was cleaned under running water, and the surface was air-dried.

#### Ceramic etch-primer

The ceramic disc surfaces in this group were conditioned with ceramic etch & primer (Monobond etch & prime, Ivoclar Vivadent, Schaan, Lichten-stein). The primer was applied to the surface, agitated for 20 s, and left intact for 40 s. Later specimens were rinsed in water and air-dried.

#### Air abrasion

Ceramic disc surfaces were air abraded using an intra-oral sandblaster ( Micro Etcher, Danville, San Ramon, CA, USA). Al_2_O_3_ with 50 𝜇m for 20 s, at 2 bar pressure. Subsequently, specimens were cleaned in distilled water and air-dried.

#### Grinding bur

The specimens in this group were roughened with a medium-grit (100 𝜇m) diamond bur (Brasseler, One Brasseler Boulevard, Savannah, GA, USA). Roughening of the surface was performed by a single operator at 45,000 rpm for an 8 s duration.

### Bonding and shear bond testing

A lower incisor metal bracket (Roth Mini Bondable, Tac Eksen, Huzhou, Zhejiang, China) and ceramic brackets (Roth E-Sapphire, Tac Eksen, Huzhou, Zhejiang, China) were bonded onto the ceramic specimen following a standardized protocol and processed by a single operator. The lower incisor brackets with − 10 torque, 00 angulation, and 2.55 mm width were bonded using a light cure adhesive composite (XT, 3 M Unitek, St. Paul, MN, USA). Two layers of silane coupling agent (RelyX ceramic primer, 3 M ESPE, St. Paul, MN, USA) were applied and dried for 60 s. The adhesive composite was applied to the bracket base, placed over the ceramic disk with manual pressure to release excess material. Subsequently, the excess was removed with a periodontal probe. Composite resin was polymerized by 1200 mW/cm2 LED light (Bleuphase, Ivoclar Vivadent, Schaan, Lichtenstein) for 20 s.

**Fig. 2:**
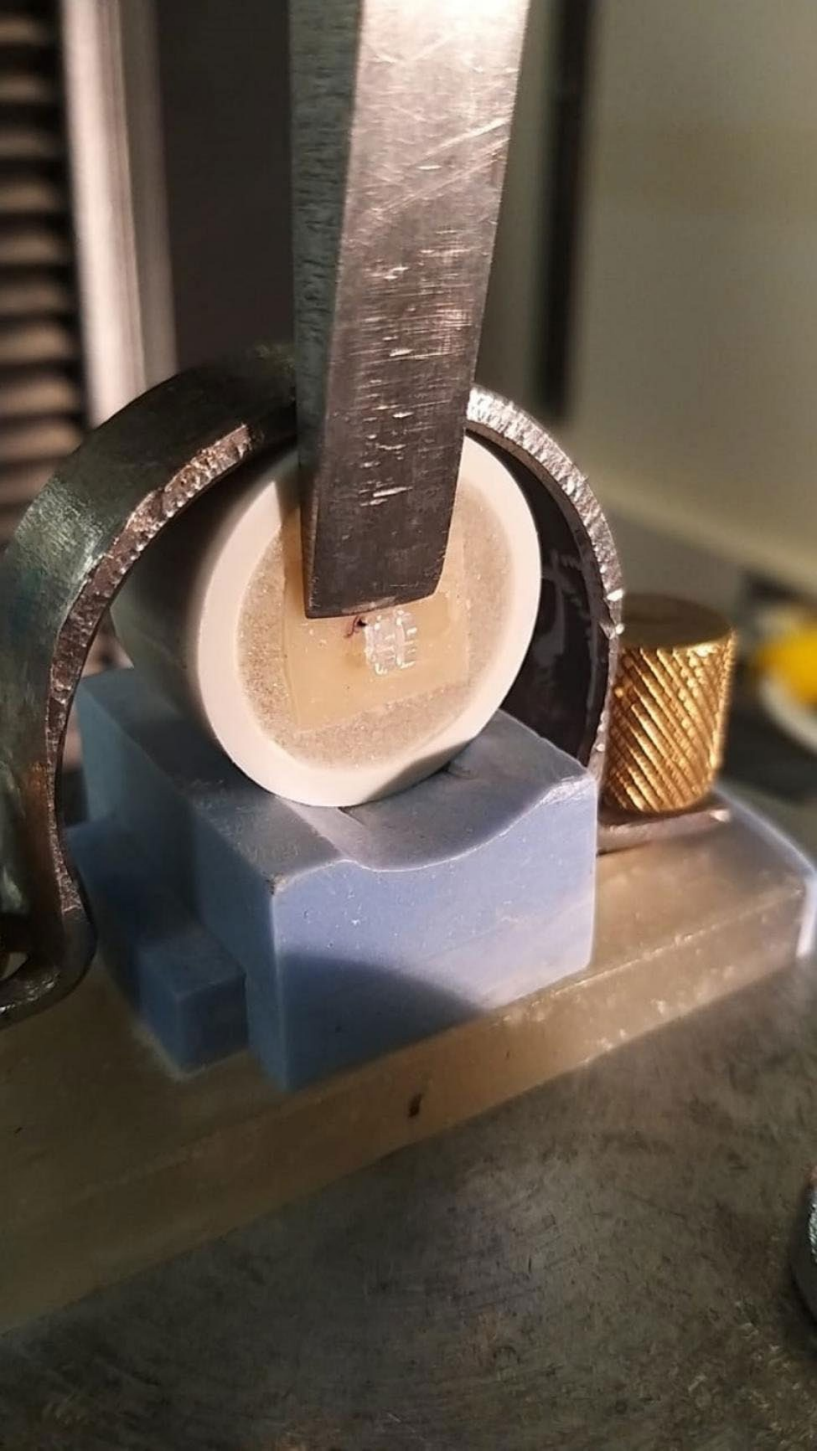
Ceramic bracket Shear bond strength evaluation with Universal testing machine

Shear bond testing was conducted according to ISO/TS 11405:2015 specification. Specimens with bonded composite restoration were stored in 37^0^ C distilled water for 24 h, followed by 10,000 thermal cycles (Thermocycler, SD Mechatronik, Feldkir-chen-Westerham, Germany) between 5 and 55^0^ C with a 30 s dwell time. Subsequently, the bonded composite–dentin interface was subjected to shear stress with a 200-µM chisel-shaped head with a ramp rate of 1 mm/min (Fig. 2). The maximum load at fracture was recorded in Newton (N), which was divided by the bracket’s base surface area to convert to MegaPascal (MPa) values.

The debonded interface was examined under a digital microscope (Hirox, Hackensack, NJ, USA) at ×25 magnification to categorize the types of failures. The adhesive remnant index (ARI) was used as a criterion to classify failure types [21]. Index 0: no adhesive on ceramic discs, Index 1: less than 50% of the adhesive on ceramic discs, Index 2: more than 50% of the adhesive on ceramic discs, Index 3: all adhesive resin still adhered to ceramic discs.

### Statistical analysis

Statistical analysis was performed using SPSS 22.0 software (IBM Corporation, Armonk, NY, USA). The data were evaluated by one-way ANOVA and Tukey HSD post-hoc tests. The level of statistical significance was 0.05.

## Results

Table 2 presents the mean and standard deviations of shear bond strength values (MPa) for ceramic and metal brackets. In most ceramic samples, ceramic brackets had higher SBS values than metal brackets. Control groups devoid of surface treatment had the lowest SBS values across all ceramic samples. The SBS values for control ceramic brackets were 6.08 (0.11) MPa, 3.95 (0.09) MPa, 6.18 (0.18) MPa, and 3.81 (0.14) MPa for LD-CAD, PIC, ZLS, and 5YZP ceramic groups, respectively. Corresponding values for metal brackets were 4.58 (0.20) MPa, 4.32 (0.16) MPa, 4.68 (0.18) MPa, and 4.28 (0.15) MPa, respectively. The LD-CAD and ZLS ceramic samples displayed higher SBS values than the PIC and 5 YZP ceramic groups in the majority of surface treatment protocols. HFA etching significantly enhanced SBS in LD-CAD for ceramic and metal brackets at 12.02(0.64)Mpa, and 9.25(0.44) Mpa, correspondingly. Ceramic and metal bracket SBS values with HFA etching also substantially increased in ZLS and PIC samples at 12.85(0.52)Mpa and 10.26 (0.55) Mpa; 9.19 (0.39) Mpa and 9.20 (0.40) Mpa, respectively. The 5YZP ceramic samples showed a lesser benefit from HFA etching, with SBS values for the ceramic bracket at 6.39 (0.41) Mpa. However, MEP and AL2O3 air abrasion surface treatments in 5YZP ceramic samples enhanced the bond strength compared to other surface treatment protocols in both ceramic brackets and metal brackets, with corresponding values of 8.95 (0.39) MPa, 7.97 (0.45) MPa, 7.95 (0.32) MPa, and 5.70 (0.31) MPa, respectively. Surface roughening with a medium grit diamond bur had the lowest SBS values across all the tested ceramic groups.

**Table 2:** Mean (SD) shear bond strength of Metal and Ceramic Bracket groups

Group	Bracket type	Control	MEP	HFA	Air Abrasion	Bur rough
LD-CAD	Ceramic	6.08(0.11)	12.02(0.64)	13.17(0.26)	10.59(0.29	7.63(0.49)
	Metal	4.58(0.20)	9.25(0.44)	10.51(0.45)	6.83(0.27)	4.85(0.27)
PIC	Ceramic	3.95(0.09)	8.86(0.48)	9.19(0.39)	5.46(0.28_	4.29(0.32)
	Metal	4.32(0.16)	8.15(0.45)	9.20(0.43)	5.38(0.34)	4.82(0.42)
ZLS	Ceramic	6.18(0.18)	10.84(0.30)	12.85(0.52)	9.60(0.93)	7.72(0.56)
	Metal	4.68(0.18)	9.76(0.28)	10.26(0.55)	6.41(0.28)	5.29(0.31)
5 YZP	Ceramic	3.81(0.14)	8.95(0.39)	6.39(0.41)	7.95(0.32)	4.07(0.27)
	Metal	4.28(0.15)	7.97(0.45)	7.00(0.32)	5.70(0.31)	4.83(0.39)

All the ceramic samples in the ceramic bracket groups evaluated in the study showed a significant effect of various surface treatments on shear bond strength with a p-value of 0.05 (Table 3). Similarly, the shear bond strength of metal orthodontic brackets to different ceramic samples showed significant differences with different surface treatments (p 0.05) (Table 3).

**Table 3:** One-Way Analysis of Variance of Mean shear bond strength of Metal and Ceramic Brackets groups

Group	Bracket	Source	SS	MS	F	*p*
LD-CAD		Between Groups	354.249	88.562	811.840	0.000*
	Metal	Within Groups	7.393	0.164		
		Total	361.641			
		Between Groups	276.330	69.082	581.606	0.000*
	Ceramic	Within Groups	5.345	0.119		
		Total	281.675			
PIC		Between Groups	220.650	55.163	470.145	0.000*
	Metal	Within Groups	5.280	0.117		
		Total	225.930			
		Between Groups	187.220	46.805	323.547	0.000*
	Ceramic	Within Groups	6.510	0.145		
		Total	193.730			
ZLS		Between Groups	227.919	56.980	178.484	0.000*
	Metal	Within Groups	14.366	0.319		
		Total	242.285			
		Between Groups	265.401	66.350	549.456	0.000*
	Ceramic	Within Groups	5.434	0.121		
		Total	270.835			
5 YZP		Between Groups	210.719	52.680	496.693	0.000*
	Metal	Within Groups	4.773	0.106		
		Total	215.492			
		Between Groups	92.979	23.245	196.515	0.000*
	Ceramic	Within Groups	5.323	0.118		
		Total	98.302			

*Significance(p) < 0.05 level

A Tukey HSD post hoc analysis (Table 4) in ceramic bracket groups revealed statistically significant differences between all the tested surface treatments except control and diamond bur grinding; MEP and HFA etching in PIC samples. Similarly, an insignificant difference was observed in 5YZP ceramic between the control and diamond bur grinding groups. A Tukey HSD post hoc analysis (Table 4) of the metal bracket group recorded significant differences between the majority groups except for control and diamond bur grinding in PIC and 5 YZP ceramic samples.

**Table 4:** Tukey HSD Post-hoc analysis of Mean Micro-shear bond strength amongst Metal and Ceramic Bracket groups

Group	(I) Group	(J) Group				
		Control	MEP	HFA	Airabrasion	Grind bur
LD-CAD	Control		0.000*/0.000*	0.000*/0.000*	0.000*/0.000*	0.417*/0.000*
	MEP	0.000*/0.000*		0.000*/0.000*	0.000*/0.000*	0.000*/0.000*
	HFA	0.000*/0.000*	0.000*/0.000*		0.000*/0.000*	0.000*/0.000*
	Air abrasion	0.000*/0.000*	0.000*/0.000*	0.000*/0.000*		0.000*/0.000*
	Grind bur	0.417/0.000*	0.000*/0.000*	0.000*/0.000*	0.000*/0.000*	
PIC	Control		0.000*/0.000*	0.000*//0.000*	0.000*/0.000*	0.038/0.173
	MEP	0.000*/0.000*		0.399/0.399	0.000*/0.000*	0.000*/0.000*
	HFA	0.000*/0.000*	0.399/0.399		0.000*/0.000*	0.000*/0.000*
	Air abrasion	0.000*/0.000*	0.000*/0.000*	0.000*/0.000*		0.018*/0.000*
	Grind bur	0.038/0.173	0.000*/0.000*	0.000*/0.000*	0.018*/0.000*	
ZLS	Control		0.000*/0.000*	0.000*/0.000*	0.000*/0.000*	0.002*//0.000*
	MEP	0.000*/0.000*		0.020*/0.000*	0.000*/0.000*	0.000*/0.000*
	HFA	0.000*/0.000*	0.020*/0.000*		0.000*/0.000*	0.000*/0.000*
	Airabrasion	0.000*/0.000*	0.000*/0.000*	0.000*/0.000*		0.000*/0.000*
	Grind bur	0.002*/0.000*	0.000*/0.000*	0.000*/0.000*	0.000*/0.018	
5 YZP	Control		0.000*/0.000*	0.000*/0.000*	0.000*/0.000*	0.006/0.378
	MEP	0.000*/0.000*		0.000*/0.000*	0.000*/0.000*	0.000*/0.000*
	HFA	0.000*/0.000*	0.000*/0.000*		0.000*/0.000*	0.000*/0.000*
	Air abrasion	0.000*/0.000*	0.000*/0.000*	0.000*/0.000*		0.000*/0.000*
	Grind bur	0.006/0.378	0.000*/0.000*	0.000*/0.000*	0.000*/0.000*	

* Metal Bracket / Ceramic Bracket, Significance(p) < 0.01 level

Table 5 presents the failure type distribution. Failure types in control were pre-dominantly ARI − 0, indicating adhesive failures at the resin-ceramic interface. LD-CAD and ZLS ceramic groups with MEP and HFA surface treatments displayed largely ARI-1 and ARI-2 failures. The majority of PIC and 5 YZP ceramic groups with MEP surface treatment had ARI-I failures. However, diamond-bur abrasion surface treatment across ceramic groups had ARI-0 major failures.

**Table 5:** Frequency distribution of adhesive remnant index (ARI) scores in ceramic brackets

ARI Scores	LD-CAD				PIC				ZLS				5 YZP			
	0	1	2	3	0	1	2	3	0	1	2	3	0	1	2	3
**Ceramic bracket**																
Control	5	3	2	0	7	3	0	0	6	3	1	0	7	3	0	0
MEP	0	5	2	3	0	4	2	4	0	6	3	1	1	4	3	3
HFA	0	5	3	2	2	4	2	2	0	5	3	2	4	3	2	1
Al_2_O_3_ Air abrasion	3	4	2	1	5	3	1	0	4	5	1	0	2	6	2	0
Bur roughening	4	4	2	0	6	3	0	0	5	5	0	0	7	3	0	0
**Metal bracket**																
Control	4	2	2	0	5	5	0	0	4	2	2	0	6	4	0	0
MEP	0	6	3	1	0	3	3	4	0	4	3	3	2	4	2	2
HFA	0	5	3	2	2	4	2	2	0	4	2	4	2	3	2	3
Al_2_O_3_ Air abrasion	3	5	2	0	5	5	0	0	5	3	2	0	8	2	0	0
Bur roughening	4	6	0	0	7	3	0	0	6	3	1	0	6	3	1	0

## Discussion

The dentist frequently encounters orthodontic patients with existing ceramic restorations. Obtaining optimum bond strength for recently introduced esthetic CAD-CAM ceramic restorations is clinically challenging. The recommended bracket bonding force is 6–8 MPa for efficient clinical application [6, 22]. Ozden AN, et al. [23] suggested that an SBS value exceeding than 13 MPa predisposes the ceramic surface to fracture during debonding of the brackets. Hence, ascertaining the surface treatment protocols to achieve a favorable SBS value in different ceramic restorations is essential for clinical applications. The present study assessed the SBS of metal and ceramic orthodontic brackets on different CAD/CAM ceramic surfaces after various surface treatments. Results of the study showed that ceramic material and surface treatment influenced shear bond strength. The various surface treatments affected the shear bond strength of the ceramic surface, which disproved the null hypothesis.

The study results revealed that various surface treatments of CAD-CAM ceramic materials enhanced the SBS of both metal and ceramic brackets. The bond strengths of control groups for ceramic brackets to LD-CAD, PIC, ZLS, and 5 YZP were low at 6.08(0.11)MPa, 3.95(0.09)MPA, 6.18(0.18)MPa, and 3.81(0.14)MPa, respectively. Corresponding values for the control group for metal brackets were also low at 4.58(0.20)MPa, 4.32(0.16)MPa, 4.68(0.18)MPa, and 4.28(0.15)MPa. The study results also revealed that ceramic brackets had better SBS strength than stainless steel across all the ceramic surfaces. The only exception was that the SBS values of stainless-steel brackets for 5YTZP zirconia surfaces were moderately greater than ceramic brackets. There is no consensus in the literature about the higher bond strength potential between ceramic and stainless-steel brackets. Al-Hity R [24] Elsaka SE et al. [16] reported similar results of greater SBS with ceramic brackets, while Pinho M et al. [25], reported a higher bond strength with stainless-steel brackets. Enhanced SBS in ceramic brackets could be due to their larger adhesive base area than metal brackets. Furthermore, the ceramic bracket adhesive surface provides micromechanical retention due to randomly oriented polycrystalline alumina or glass particles.

Hydrofluoric acid surface etching enhanced the bond strength of both metal and ceramic brackets in LD-CAD, PIC, and ZLS ceramics. IPS-Emax CAD is comprised of 58% silica, lithium-meta silicate, disilicate, and phosphate crystals, and 10% zirconia crystals. During the two-stage crystallization process, lithium meta silicate crystallizes (0.2–1.0 mm size), and subsequent heating under vacuum leads to the formation of fine lithium-disilicate crystals (70 vol% of 1.5 mm grain size) within a glassy matrix [26]. The manufacturers polymerized pre-sintered porous ceramics immersed in resin monomers to create PICNs. Hence, it has a dual-network structure of the ceramic skeleton and polymer phases [27]. Zirconia-reinforced lithium silicate (ZLS) is produced by reinforcement of lithium meta silicate (Li2SiO3) glass ceramic with approximately 10% zirconium dioxide (ZrO2). The surface treatment of these ceramics modifies the surface microstructure by dissolving both the glassy and polymer phases. The resultant microporosity over the ceramic surface enhances the surface area, wettability, and surface energy of the substrate and micro-mechanical interlocking [28]. Surface modification from etching varies in different ceramic materials due to divergences in composition and crystalline and vitreous phase distributions. HFA etching of glass ceramics, besides improving micro-mechanical retentive features, also fosters hydroxyl formation for silane-facilitated bonding [29].

Straface A [30] reported the same outcome of increased shear bond strength by hydro-fluoric acid etching in LD-CAD, PIC, and ZLS ceramics. Likewise, Elsaka SE [16] found that HFA etching improved bond strength in PIC ceramics. PIC comprises the dominant inorganic structure (58–63%) of SiO2. It allows the selective dissolution of an amorphous ceramic structure [31], and silane content within the PIC improves bond strength [32]. The moderately low shear bond strength observed in PIC ceramics was analogous to Avram LT et al. [33]. They attribute the lesser bond strength post-HFA etching to an insignificant alteration in the polymer component and weak interatomic bonds. Acid etching does not affect zirconia because they are non-silica-based ceramics. We found that the bond strength in both ceramic and metal brackets for 5YTZP was not significantly improved. Wang Y et al. [34] recorded similar results of higher bond strength after HFA etching and universal adhesive treatment in glass ceramics groups, including LDS-CAD and ZLS ceramics in contrast to zirconia. Smielak B et al.[35] suggested a 9.5% HF concentration, and 15 min etching duration. Meanwhile, Alghazzawi TF et al.[36] found that the temperature of the HFA solution affected the etching efficiency. Sriamporn T et al. [37] reported that immersing zirconia in 9.5% HF at 80^0^ C for 1 min or at 25^0^ C for 1 h led to surface roughness. However, surface modification with a higher concentration or higher temperature of HFA for a longer duration in intra-oral conditions is not feasible.

Clinicians prefer the all-in-one surface conditioning, Monobond Etch and Prime (MEP), which comprises both acid etching and salinization steps. Moreover, it prevents iatrogenic accident, leading to deep soft tissue injuries and bone necrosis with the HFA intra-oral application. MEP contains ammonium polyfluoride for etching and trimethoxypropyl methacrylate for silanization. MEP surface conditioning showed a significant improvement in shear bond strength in metal and ceramic brackets across all ceramic groups. SBS values for all the groups were marginally less than HFA etching, except for the ceramic brackets on zirconia ceramic. El-Damanhoury HM [38], and Prado M [39], reported a similar observation of higher SBS values with the HFA etching protocol in comparison to MEP surface conditioning. They attribute it to the lesser surface roughness of MEP than conventional HFA. Although González-Serrano C [40] recorded marginally lower SBS values for MEP conditioning in lithium disilicate ceramics, the difference was insignificant after 24 h. Murillo-Gómez F et al. [41] observed that HFA produced an aggressive etching morphology pattern in LDS-CAD and PIC ceramics, with a lower Si/C ratio than MEP. They recommend MEP treatment as an alternative to aggressive HFA treatment to avoid internal alterations to ceramic structural configuration. Maier et al. [42] also reported that the mean bond strengths of MEP groups in glass ceramics did not differ significantly from HF-etched and silanized specimens. They attribute it to the lower likelihood of moisture contamination between the etching and silanization steps. In addition, they attribute it to the sedimentation of silica fluoride over ceramic surfaces after HFA etching. Dönmez MB et al. [43] also recorded higher SBS values with HFA surface treatment in lithium disilicate glass ceramics. They advocate MEP as an effective method to obtain adequate bond strength values. The study results indicate that the use of MEP for zirconia surface conditioning has significant potential to improve orthodontic bracket SBS strength. MEP conditioning achieved the highest SBS values for zirconia samples for metal and ceramic brackets at 7.97(0.45) MPa and 8.95(0.39) MPa respectively. Franz A et al. [44] corroborated our findings with higher SBS values with zirconia MEP conditioning and sustained bond strength during artificial aging. They assume that ammonium polyfluoride could cause surface modifications.

The study indicated that air abrasion with 50 m aluminum-oxide particles enhanced the bond strengths of metall and ceramic brackets for all the tested ceramic groups. It significantly improved the bond strength in zirconia groups in both ceramic and metal brackets. Air abrasion with aluminum oxide particles at high pressure creates micro-retentive surface morphology [45]. Yang et al. [46] observed from XPS analysis that sandblasting effectively removed silicone residues and salivary contaminants from the ceramic surfaces. Acid monomer components in MDP primers interact with zirconia oxide, facilitated by improved surface energy and wettability post-air abrasion [47]. Özarslan MM et al. [48] described the equivalent benefit of HPA etching and aluminum-oxide sandblasting to promote better bond strength in lithium disilicate and PIC ceramics. Byeon SM, et al. [49] used Al2O3 sandblasting to enhance zirconia and metal bracket bonding strength. Study results confirmed that surface roughening with a medium-grit bur was inadequate for enhancing bond strength across all the ceramic groups. Dilber et al. [50] corroborated this observation; they recommended additional surface conditioning with HFA etching or silica coating after surface grinding with a diamond bur. Schmage et al. [51] observed that roughening with a diamond bur without silane application resulted in reduced bond strength compared to HFA etching and sandblasting. They believe that surface roughness resulting from sandblasting and diamond roughening can damage ceramic surfaces.

The control groups failure type in both metal and ceramic brackets amongst all the groups were predominantly adhesive, indicating the inability to achieve a strong bond with ceramic surfaces. ARI scores in LD-CAD, PIC, and ZLS ceramics with HFA and MEP surface treatment were largely in score 1, with less than half of the luting material remaining on the ceramic surface. Most of the failures were caused by cohesive failures within the luting resin, rather than by failures within the ceramic substrate. Due to the strong mechanical interlocking, chemical bonding, and better flexural strength of LD-CAD (530 MPa), PIC (137 MPa), and ZLS (420 MPa), failures occurred within the composite resin. However, PIC groups with abrasion and bur roughening failures were adhesive in nature. The zirconia groups observed adhesive failure at a resin-zirconia interface, indicating lower bond strengths between resin and zirconia substrates.

The clinical significance of the study includes the dentist’s need to choose an effective ceramic surface conditioning method depending on the composition and structural properties of the ceramic material. Metal and ceramic orthodontic brackets bond strength to ceramic surfaces devoid of surface treatment is inadequate for clinical application. Hence, performing suitable surface treatment before orthodontic bracket application is essential. HPA etching and silane application for LD-CAD, PIC, and ZLS ceramics resulted in sufficient improvement in SBS values. MEP application appears to be an effective alternative to conventional HPA etching in both glass ceramic groups and zirconia ceramics. Our study’s findings can also be useful in understanding the clinical behavior of lingual orthodontic appliances [52] and aligner attachments [53]. The various surface treatments affect the surface morphology, structural network, and content of the ceramic substrate. HFA etching produces deeper and larger roughness in glass ceramic groups, while it also induces an increase in monoclinic phase content within zirconia ceramics. Therefore, further studies are recommended to evaluate the impact of various surface treatments on the biomechanical and clinical performance of different ceramic materials, as well as their relevance in lingual brackets. Furthermore, studies are required to evaluate the sustainability of bond strength achieved through different surface treatments after artificial and hydrothermal aging. Further research into the interaction of ammonium polyfluoride in MEP with yttrium-stabilized zirconium oxide ceramics would be of great significance.

As with all in vitro studies, the limitations of this research include the challenge of replicating the complex oral environment. Various factors, such as changes in pH, bacterial flora, temperature variations, and masticatory stress within the oral environment, can influence the outcome. Hence, the study result needs to be corroborated by a clinical study.

## Conclusion

Within the limitations of this in vitro study, the following conclusions can be drawn: Glazed surfaces, without additional surface conditioning, presented unsatisfactory SBS values for orthodontic brackets in clinical applications. Ceramic materials’ structural and crystalline properties influenced the efficiency of each surface treatment protocol in improving SBS values. All glass-ceramic groups, including LD-CAD, PIC, and ZLS ceramics, showed high shear bond strength after HFA etching and silane application. Using MEP application as a surface treatment is a viable alternative to HFA etching for all ceramic groups, especially for enhancing SBS values in zirconia ceramics. The bond strength on LD-CAD and ZLS ceramic surfaces was significantly improved with the use of air abrasion and silane application.

## Data Availability

The data analyzed in this study are available from the corresponding author on reasonable request.

## Abbreviations

LD-CAD: Lithium disilicate CAD
PIC: Polymer-infiltrated ceramic
ZLS: Zirconia-reinforced lithium silicate glass ceramic
5YTZP: 5 mol% yttria-stabilized zirconia polycrystalline ceramic
HFA: Hydrofluoric acid
